# Multi-center, randomized, double-blind, placebo-controlled, exploratory study to evaluate the efficacy and safety of HAD-B1 for dose-finding in EGFR positive and locally advanced or metastatic NSCLC subjects who need Afatinib therapy: Study protocol clinical trial (SPIRIT Compliant)

**DOI:** 10.1097/MD.0000000000018735

**Published:** 2020-01-24

**Authors:** So-Jung Park, Hwi-Joong Kang, Hyung-Joon Jun, Seong-Hoon Shin, Hwa-Seung Yoo

**Affiliations:** a East-West Cancer Center, Dunsan Korean Medicine Hospital of Daejeon University, Daejeon; b Department of Internal Medicine, Kosin University Gospel Hospital, Busan, Korea.

**Keywords:** afatinib, HAD-B1, herbal medicine, non-small cell lung cancer, randomized controlled trial

## Abstract

**Background:**

In recent studies, afatinib, a second-generation inhibitor, showed superior outcomes, when compared to the first-generation of EGFR-tyrosine kinase inhibitors (TKIs), such as erlotinib and gefitinib, in patients with advanced non-small cell lung cancer (NSCLC) harboring mutations of epidermal growth factor receptor (EGFR). Patients who receive TKIs with a significant initial efficacy, inevitably experience an acquired resistance (AR) within 9 to 13 months. Traditional Korean medicine may have synergistic effects when combined with chemotherapy or radiotherapy. The purpose of this trial is to assess whether afatinib plus HAD-B1 improves disease control rates (DCRs) compared with afatinib alone and to evaluate the efficacy and safety of HAD-B1 for finding the proper dose.

**Methods:**

This is a randomized, double-blind, placebo-controlled, multi-center, therapeutic, exploratory clinical trial. This trial is designed to determine whether HAD-B1 combined with afatinib results in better DCRs with less toxicity than afatinib alone. A total of 66 NSCLC patients with EGFR mutations will be randomly assigned to treatment group 1 (afatinib 40 mg/day plus HAD-B1 972 mg), treatment group 2 (afatinib 40 mg/day plus HAD-B1 1944 mg) and a control group (afatinib 40 mg/day). Afatinib combined with HAD-B1 or with a placebo will be administered to the participants for 12 weeks. The primary endpoint is a comparison of the DCRs among groups. Secondary endpoints are comparisons of the complete response (CR) and the partial response (PR) to the treatment, the stability of the disease (SD), progression free survival (PFS), time to progression (TTP), and tumor marker (CEA, NSE) and WBC differential count (LMR, NLR) and natural killer cell activity and quality of life (QOL) among groups.

**Discussion:**

The results from this clinical trial will provide evidence of efficacy and safety of HAD-B1 in EGFR positive and locally advanced or metastatic NSCLC patients who need afatinib therapy.

## 1 Introduction

Lung cancer is one of the most common malignant tumors in the world and is the leading cause of cancer-related mortality in males and females, according to statistics gathered in the United States in 2017.^[1]^ Lung cancer is divided into small cell and non-small cell lung cancer (NSCLC). NSCLC accounts for more than 85% of all lung cancer cases.^[2]^ Even with several advances in the staging, diagnostic procedures and therapeutic options, the overall prognosis has little changed for the majority of patients with the overall 5-year survival having only slightly increased over the last decade from 15.7% to 17.4%.^[3]^

Since the 2000s, the understanding of the pathogenesis of lung cancer has broadened, and now NSCLC can be classified into several subtypes according to mutations such as those involving epidermal growth factor receptor (EGFR), anaplastic lymphoma kinase (ALK), ROS proto-oncogene 1 receptor tyrosine kinase (ROS1) and programmed cell death protein 1 (PD-1). In addition, the treatment of lung cancer using these mutations has changed remarkably.^[4]^ In particular, EGFR tyrosine kinase inhibitors (TKIs), such as gefitinib and erlotinib, have been reported in a randomized-controlled phase-3 trial to prolong the progression-free survival (PFS) and to improve the quality of life (QOL) when compared to conventional platinum-based chemotherapy.^[5,6]^

Afatinib is a second-generation target anticancer drug that blocks all three ErbB family members: EGFR (ErbB1), HER2 (ErbB2), and ErbB4. It is also more advanced than the first-generation target therapeutics, gefitinib and erlotinib, which block only EGFR. Afatinib has been reported to improve overall survival significantly in comparison to conventional standard therapy.^[7]^ However, despite these improved therapeutic results, patients undergoing EGFR-TKI maintenance therapy usually experience disease progression due to secondary resistance that develops after about 9 to 13 months. For this reason, if the cure rate is to be improved, studies to find ways to overcome, or at least alleviate, the resistance to anticancer drugs that may occur over time in patients undergoing EGFR-TKI maintenance therapy are crucial.^[4]^

In patients with NSCLC, chemotherapy (CT) combined with herbal medicine therapy can reduce CT toxicity, prolong survival rate, enhance immediate tumor response, and improve the score on the Karnofsky performance status scale (KPS).^[8]^ Also, herbal medicine can be considered an efficient and safe maintenance therapy strategy for patients who show no progression after first-line chemotherapy, including those with poor QOL.^[9]^ Moreover, traditional Korean medicine, when combined with conventional cancer treatment, may improve the survival rate due to a synergism among improved QOL, enhanced immunity and reduced side effects.

HAD-B1 (Table 1) is a medical substance in traditional Korean medicine. It is composed of *Panax ginseng C.A.*, *Panax notoginseng Radix*, *Cordyceps militaris*, and *Boswellia carterii BIRDWOOD* and has been reported to have an anti-lung-cancer effect in xenograft animal model experiments using A549 lung cancer cells and A549/CR cells.^[10,11]^ Therefore, in this study, we aim to assess whether or not afatinib plus HAD-B1 can improve the DCR compared with afatinib alone and to evaluate the efficacy and safety of HAD-B1 for finding the proper dose for patients with EGFR positive and locally advanced or metastatic NSCLC.

**Table 1:**
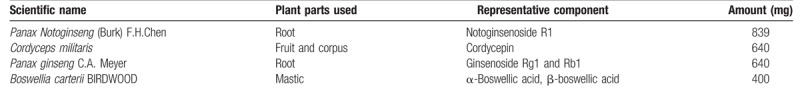
Components of HAD-B1.

## 2 Methods

### 2.1 Trial design

This study is a randomized, double-blinded, placebo-controlled trial that aims to examine the efficacy and safety of HAD-B1 for EGFR positive and locally advanced or metastatic NSCLC patients who need afatinib therapy. Also, the results will provide the proper dosage of HAD-B1 for the patients. Participants will be randomized using a ratio of 1:1:1 into treatment group 1 (afatinib 40 mg/day plus HAD-B1 2 tablets (972 mg/day) plus placebo 2 tablets, treatment group 2 (afatinib 40 mg/day plus HAD-B1 4 tablets [1944 mg]) and control group (afatinib 40 mg/day plus placebo 4 tablets). Afatinib combined with HAD-B1 or placebo will be administered to the participants for 12 weeks (Table 2).

**Table 2:**
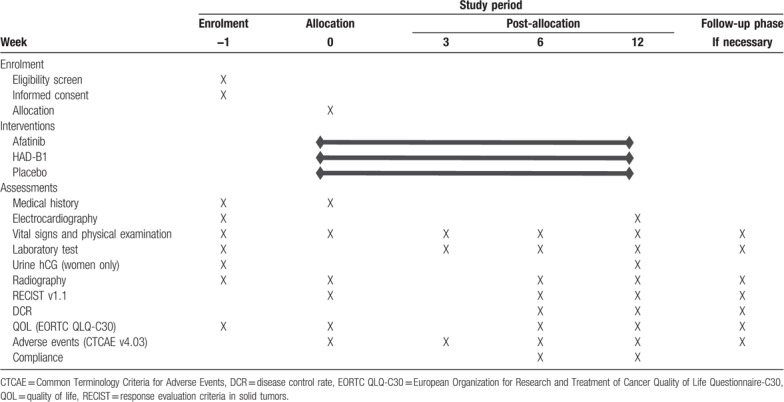
Summary of study design.

### 2.2 Recruitment and consent

**Figure 1:**
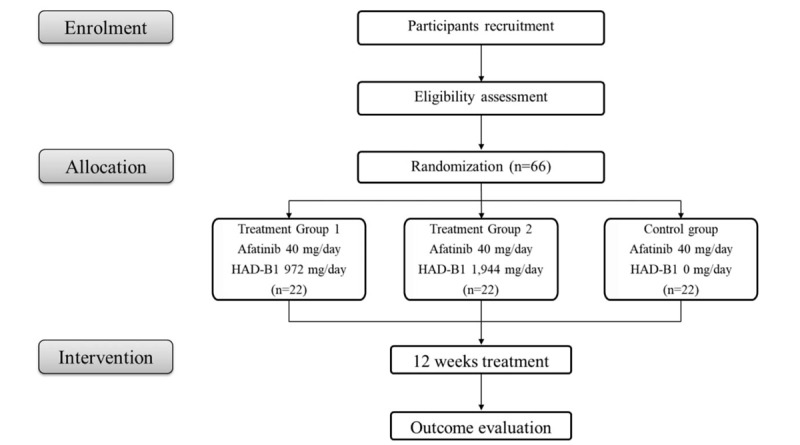
Study flow chart.

A total of 66 subjects with locally advanced or metastatic EGFR mutation-positive NSCLC who need afatinib therapy will be recruited at Kosin University Gospel Hospital, Busan National University hospital. All candidates will undergo a standardized interview and receive clinical study information about the trial. Written consent will be obtained from each participant. The purpose, procedures, and potential risks and benefits of the study will also be explained thoroughly to the participants. The participants will be able to withdraw from the study at any time without consequence. The trial covers the period from September 2018 to October 2020, including the enrolment and follow-up periods (Fig. 1).

### 2.3 Inclusion criteria

Participants meeting the following criteria will be included:

1. 19 years of age or older;
2. Diagnosed with locally advanced or metastatic NSCLC, but unable to undergo surgery or radiation therapy;
3. Histologically or cytologically EGFR-positive and requiring afatinib therapy;
4. Presence of a lesion that can be measured by using chest X-ray, computed tomography (CT) or magnetic resonance imaging (MRI) with a single diameter or two diameters;
5. Eastern Cooperative Oncology Group (ECOG) performance status score from 0 to 2;
6. Life expectancy of more than six months, as well as normal bone marrow function and solid organ function for more than 6 months:
  (a) Bone marrow: ANC ≥ 1.5 × 10^9^/L, platelet ≥10 × 10^9^/L, and hemoglobin ≥10 g/dL,
  (b) Liver function: AST/ALT levels below twice the normal upper limit,
  (c) Kidney function: creatinine levels below twice the normal upper limit;
7. Voluntarily signed written informed consent to participate in this trial.

### 2.4 Exclusion criteria

Participants meeting one or more of the following criteria will be excluded:

1. Experience with severe drug hypersensitivity to a particular drug or inability to take oral drugs;
2. Currently pregnant or breastfeeding (fertile women will be required to use adequate contraception during the study period);
3. Presence of metastatic cancer in the central nervous system or in need of concurrent therapy, such as primary site radiation therapy, chemotherapy, or immunotherapy;
4. T790 M (threonine-to-methionine amino acid change at position 790) of the EGFR kinase domain (acquired, re-biopsy) mutation-positive;
5. The presence of other serious disease:
  (a) Uncontrollable congestive heart failure or unstable angina, myocardial infarction within 1 year of study participation, uncontrollable hypertension or high-risk arrhythmia,
  (b) Inability to understand the research content due to neurological or psychiatric disorders, such as dementia and seizures,
  (c) Uncontrollable infections, such as tuberculosis and hepatitis;
6. A history of diagnosis or treatment of malignant tumors other than NSCLC;
7. Participation in research involving the combined administration of any other drug, except for afatinib, and in any other clinical trial within the previous 2 weeks;
8. A need for administration of immuno-inhibitors or platinum-based chemotherapy after a PD-L1 expression test with multiple metastases with symptoms;
9. Use of a prohibited medication during this clinical trial;
10. Unsuitable for this clinical trial as determined by the researcher.

### 2.5 Interventions

Patients in the treatment group 1 will receive afatinib (40 mg/day orally) plus HAD-B1 2 tablets (972 mg/day, bid orally) and placebo 2 tablets, those in the treatment group 2 will receive afatinib (40 mg/day orally) plus HAD-B1 4 tablets (1944 mg/day, bid orally), while those in the control group will receive afatinib (40 mg/day orally) plus placebo 4 tablets (bid orally). Afatinib, HAD-B1 and/or placebo will be administered consistently for 12 weeks; the trial may be terminated before the end of 12 weeks due to the development of unacceptable toxicities or withdrawal of the participant from the treatment.

The HAD-B1 tablets used in clinical trial will purchased from the Gyeongbang Pharmaceutical Co., Ltd. (In-cheon, Korean) and will meet the requirements of Good Manufacturing Practice (GMP). HAD-B1 is a medical substance that contains *Panax ginseng C.A.* (640 mg), *Panax notoginseng Radix* (839 mg), *Cordyceps militaris* (640 mg), and *Boswellia carterii BIRDWOOD* (480 mg) as raw material medicines is extracted for 16 hin purified water at 60°C. Then, the filtrate is concentrated under reduced pressure to obtain a dry extract of 486 mg. From this, 650 mg of one brown film-coated tablet is produced by adding excipients.^[10]^ The matching placebo will be purchased from the same manufacturer and consist of starch with no active ingredients.

### 2.6 Outcome measures

The primary outcome is a comparison of the disease control rates (DCR) among treatment groups 1 and 2 and the control group after 12 weeks compared to baseline, which will be assessed from the date of randomization to 12 weeks, as determined by chest X-ray, CT or MRI according to the RECIST criteria.^[11]^

The secondary outcomes are comparisons of the complete response (CR) and the partial response (PR) to the treatment, the stability of the disease (SD), progression free survival (PFS), time to progression (TTP), and tumor marker (CEA, NSE) and WBC differential count (LMR, NLR) and natural killer cell activity and QOL (EORTC QLQ-C30) among treatment groups 1 and 2 and the control group after 6 weeks and 12 weeks compared to baseline.

### 2.7 Safety assessments

During the study period, participants will be continuously monitored for adverse reactions. In addition, symptoms and vital signs will be recorded, routine physical examinations will be given, and complete blood tests will be done according to the National Cancer Institute Common Terminology Criteria for Adverse Events, version 4.03 (NCI CTCAE v4.03).^[12]^ These will be done before, during, and after the 12-week trial. If no adverse reactions occur, all assessments will be completed during the last visit. At each visit, participants will be questioned, and all adverse events will be recorded; all reported adverse events will be analyzed. Safety and tolerability will be assessed according to the NCI CTCAE v4.03.

### 2.8 Randomization and blinding

After informed consent has been obtained, random assignment will be performed by using a computer-generated, blocked, random-allocation sequence with a 1:1:1 ratio. Each participant will be assigned to a group according to a pre-generated randomization table and an assigned randomized number. Each participant will be assigned to treatment group 1, treatment group 2, or the control according to the allocation codes of the randomized assignment method, which will be prepared in advance. In order to maintain a double-blind trial, we will use the same formulations and properties for the placebo so that it is indistinguishable from the test drug. The allocation of unique codes for each group will be managed by the managing pharmacist, will be kept in a sealed envelope, and will not be disclosed until the end of the clinical trial.

### 2.9 Sample size calculation

The sample size was determined by considering the number of participants available, the minimum range of the efficacy assessment, and the expected dropout rate during the study period. When the first two factors were considered, the total number of participants required for each group was calculated as 15, for a total of 45. When a dropout rate of 30% was used for each group, the required number of participants was calculated as 22 (15/(1 – 0.3) = 21.43 ≒ 22). Thus, when all factors are considered, the number of participants to be registered for each group is 22, so a total of 66 patients will be recruited.

### 2.10 Statistical analysis

#### 2.10.1 Analysis group

The data obtained from the subjects of this clinical trial will mostly be divided into a safety group, a FAS (full analysis) group and a PP (per protocol) group. The safety group will include participants who took the HAD-B1 or the placebo at least once. The FAS group will include participants who received the clinical trial medication, followed by one or more measurements of the primary outcome. Participants in FAS group who violate major inclusion, exclusion criteria or have never taken drug for clinical trial can be excluded. The PP group will include participants from among the FAS group who successfully completed this clinical trial according to the trial plan. Participants in PP group who violate test plan or take forbidden drug or do not obey a schedule can be excluded.

### 2.11 Principles of interpretation of results

The main analysis will be the FAS group analysis for the validity evaluation. The PP group analysis will be carried out separately to evaluate whether or not any differences from the FAS analysis results exist. For the FAS group, if a defect value happens at any point or a dropout occurs before the clinical trial ends, the most recent data will be analyzed as if it were obtained at that point in time (Last Observation Carried Forward Analysis). The SG analysis will be conducted to assess safety. All statistical analyses will be performed with SPSS version 21.0; no interim analyses will be performed.

### 2.12 Characteristics and health status of each group

In order to verify whether or not statistical differences in characteristics and/or health status exist among treatment group 1, treatment group 2, and the control group, we will calculate the average, standard deviation, minimum value and maximum value for continuous data, and we will compare the average values for the three groups by using the t-test. For the purpose of group comparisons, we will use the chi-squared test or Fisher's exact test for categorical data.

### 2.13 Analysis of first outcome variables

Descriptive statistics (number in each group, average, standard deviation, minimum value and maximum value) will be used for each variable affecting the DCR (CR, PR, and SD), and the results for the two test groups and the control at the last visit versus the results at baseline will be presented. The statistical significance of any difference found between test groups and the placebo group will be analyzed using chi squared test. If any difference among three groups (2 test groups and 1 placebo group) is found, Bonferroni or Duncan method will be used. Chi squared test between placebo group and treatment group 1, placebo group and treatment group 2 will be additionally conducted.

### 2.14 Analysis of second outcome variables

Descriptive statistics (number in each group, average, standard deviation, minimum value and maximum value) will be used to describe differences in the CR, PR, SD, PFS, TTP, tumor marker (CEA, NSE), WBC differential count (LMR, NLR), natural killer cell activity and QOL (EORTC QLQ-C30) between the two test groups and the control, and any differences between either of the two test groups and the control at the last visit versus baseline will be presented. The statistical significance of any difference found between test groups and the placebo group will be analyzed using chi squared test. If any difference among three groups (2 test groups and 1 placebo group) exist, Bonferroni or Duncan method will be used. Chi squared test between placebo group and treatment group 1, placebo group and treatment group 2 will be additionally conducted. The statistical significance of any variable in any group will be tested by using the paired *t* test or the Wilcoxon signed rank test.

### 2.15 Safety assessment

Adverse reactions, symptoms and vital signs, and the results of routine physical examinations and complete blood analyses will be recorded at baseline prior to administration of the drug, at intermediate visits and at the final visit. Adverse events will be assessed throughout this clinical trial.

### 2.16 Data management

Subject clinical trial data is collected using an electronic data capture (EDC) system. All data is entered into the e-CRF according to the e-CRF instructions provided by the sponsor. The sponsor or its agent performs verification with the supporting documentation for the completeness of the data. If data is missing or inconsistent, use the EDC system to resolve it with the principal in charge. The investigator shall digitally sign the locked e-CRF after verifying the entered data. Records of the identity of all subjects are kept confidential and subject identity is kept confidential even when the results of clinical trials are published. However, sponsors, monitors, IRB committees and directors of the KFDA involved in the study may view the subject's records for the purpose of monitoring, reviewing and managing the progress of the study. All information should be kept confidential and in place with confidentiality facilities and management standards. In addition, all documents related to clinical trials, such as electronic case records, shall be recorded and distinguished by subject identification code, not subject's name.

## 3 Discussion

Afatinib is an available drug that is administered orally. It is an ErbB family blocker that, in comparison with the first-generation EGFR-TKIs, irreversibly and selectively blocks signaling from all homodimers and heterodimers formed by EGFR and by the erb-b2 receptor tyrosine kinase 2 (HER2/ERBB2), HER3/ERBB3, and HER4/ERBB4 receptors.^[13,14]^ Recently, three studies on the role of afatinib have been published: a combined analysis of LuxLung 3 and 6^[15]^ and analyses of LuxLung 7^[16]^ and LuxLung8.^[17]^ In LuxLung 3 and 6, afatinib showed superiority over the first-generation EGFR-TKIs: only afatinib among EGFR-TKIs improved the overall survival in comparison with the platinum-based chemo doublet. In LuxLung 7, afatinib was compared with gefitinib in a phase 2 B, open-label, randomized, controlled trial of EGFR mutation-positive NSCLC in treatment-naive patients and showed significant improvement in progression-free survival (PFS) and time-to-treatment failure. In LuxLung 8, afatinib was compared with erlotinib in a phase 3, open-label, randomized, controlled trial involving patients with a stage IIIB or IV squamous cell carcinoma who had progressed after at least four cycles of platinum-based chemotherapy and who had shown significant improvements in PFS and OS. Despite remarkable outcome results, patients receiving EGFR-TKIs usually developed secondary resistance after approximately 9 to 13 months (median PFS: 9–12 months) and showed major adverse events grade 3 or higher, including diarrhea, rash or acne, and stomatitis or mucositis.^[16–18]^

Complementary alternative medicine is becoming more popular in Korea for the treatment of patients with cancer because traditional Korean medicine may have synergistic effects when combined with chemotherapy or radiotherapy.^[19]^ One systematic review reported that herbal medicine may increase efficacy and reduce toxicity when taken in combination with EGFR-TKI by patients with advanced NSCLC.^[20]^ According to the previous study, HAD-B1 suppressed the growth of solid tumors as compared with the vehicle or the cisplatin-treated control group in an A549 cell xenograft mouse model.^[10]^ Based on this result, a well-designed, randomized, controlled trial is needed to identify the efficacy of HAD-B1 plus afatinib in the treatment of EGFR-positive patients with locally advanced or metastatic NSCLC who need afatinib therapy, which is the motivation for this trial. If this trial is successful, it should provide high-quality evidence for the efficacy and safety of using HAD-B1 plus afatinib for clinical treatment of patients with NSCLC. Our hypotheses are as follows: The use of HAD B1 should improve the efficacy of afatinib in the treatment of EGFR-positive patients with locally advanced or metastatic NSCLC and should reduce the toxicity associated with afatinib. Moreover, this trial may provide clinical evidence supporting and advancing the use of integrative cancer therapy in the treatment of patients with locally advanced or metastatic NSCLC.

## Author contributions

**Conceptualization:** So-Jung Park, Hwi-Joong Kang, Hyung-Joon Jun.

**Funding acquisition:** Hwa-Seung Yoo.

**Supervision:** Seong-Hoon Shin.

**Visualization:** So-Jung Park, Hwi-Joong Kang, Hwa-Seung Yoo.

**Writing – original draft:** So-Jung Park, Hwi-Joong Kang.

**Writing – review & editing:** So-Jung Park, Hwi-Joong Kang.
